# The increase of plasma galectin-9 in a patient with insulin allergy: a case report

**DOI:** 10.1186/1476-7961-8-12

**Published:** 2010-08-11

**Authors:** Haorile Chagan-Yasutan, Beata Shiratori, Umme Ruman Siddiqi, Hiroki Saitoh, Yugo Ashino, Tomohiro Arikawa, Mitsuomi Hirashima, Toshio Hattori

**Affiliations:** 1 Department of Emerging Infectious Diseases, Graduate School of Medicine, Tohoku University, Sendai, Japan; 2 Department of Immunology and Immunopathology, Kagawa University, Takamatsu, Japan

## Abstract

Allergic reaction to insulin is known to be associated with eosinophilia and hyper IgE. Recent report showed that eosinophilia is related with the increased synthesis of galectin-9 (GAL-9) and osteopontin (OPN). Here, we examined plasma levels of GAL-9 and OPN first time in a case of 65-year old patient with insulin allergy. Insulin aspart & insulin aspart 30 mix were given to the patient and an elevation of the eosinophil count (8440/μl, 17.6 fold) and a moderate increase of IgE (501 U/ml, reference range: 10-350 U/ml), eotaxin-3 (168 pg/ml, 2 fold), histamine (0.95 ng/ml, 5.3 fold) were found 33 days later. The plasma levels of GAL-9 and OPN were 22.5 and 1.7 fold higher than the cut-off point, respectively. After one month cessation of insulin therapy, elevations of the eosinophil count (3,480/μl; 7.3 fold), and OPN (1.4 fold) still occurred but the GAL-9 levels became normal. Therefore, we noted the increases of GAL-9 and OPN in plasma for the first time in a patient with insulin allergy and propose that GAL-9 reflects the conditions of allergy more accurately.

## Background

Allergic reaction to insulin is known to be associated with eosinophilia and hyper IgE [1]. We studied novel pro-inflammatory molecules such as galectin-9 (GAL-9) and osteopontin (OPN) in a patient with insulin allergy because the involvement of these molecules in eosinophilia has been recently proposed [2,3].

OPN is a glycoprotein believed to be involved in Th1 inflammation in various infectious diseases including HIV as we described previously [4]. Recently, it was reported that OPN is synthesized by eosinophils and was elevated in bronchoalveolar lavage (BAL) fluid of asthma patients [2]. GAL-9 is a member of the galectin family of thiol-dependent lectins, and positive GAL-9 staining was observed in drug injured liver tissue [3]. Recently, it was reported that GAL-9 treated NOD mice had decreased populations of Th1 cells and less leukocyte infiltration in islets than the control group indicating that GAL-9 inhibits autoimmune diabetes in NOD mice [5].

Here, we measured the plasma OPN and GAL-9 levels in a patient with insulin allergy for the first time. In addition, we also investigated the levels of soluble interleukin2 receptor (sIL-2R), eotaxin-3 and histamine, which are known to be elevated in patients with eosinophilia [6-8].

## Case presentation

A 65-year old man was referred to our department because of persistent fever on Feb. 2, 2009. The patient was diagnosed as type 2 diabetes mellitus 10 years earlier and was treated with the anti-diabetic drug metformin. He had been suffering from moderate fever since Dec. 10, 2008 and was admitted to a nearby hospital on Dec. 30. After hospitalization, various antibiotics were given due to the elevation of CRP (Table 1). Insulin aspart & insulin aspart 30 mix were also prescribed on Jan. 10, 2009 because HbA1c was also elevated. A sudden increase of the eosinophil count (Table 1) was noted on Jan. 13 and the patient was referred to our hospital for further evaluation on Feb. 2 although the fever had been subsiding. All the antibiotic drugs, but not the insulin, were discontinued due to the eosinphilia. The patient complained of generalized itching, and a rash on the back was noted. Lymph node swelling was not found and parasites were not detected in the feces. Laboratory findings showed elevations of numbers of leukocytes and eosinophils associated with the increase of inflammatory markers (Table 1). Serum IgE levels were slightly elevated. CT scan showed pleural and pericardial effusion. Bone marrow examination showed only marked eosinophilia with normal development. On Feb. 13, a further increase in the eosinophil count was noted and the insulin described above was changed to human insulin. Subsequently, the itching disappeared and the laboratory findings also became normal (Table 1). Finally, according to the above findings, the patient was diagnosed as allergy to insulin aspart & insulin aspart 30 mix.

**Table 1:** Laboratory data

Variable	Ref. Range	30-Dec-08	13-Jan-09	2-Feb-09 (on admission)	16-Feb-09	9-Mar-09	18-May-09 (on OPD)
**WBC(/μl)**	3200-9600	8670	11360	20100	23500	9400	5700
**Neu(%)**	31-65	71	58	34	14	45	52
**Lymph(%)**	18-51	12.2	16	16	23	13	35
**Mono(%)**	1-12	6.9	9	3	4	5	9
**Eosino(%)**	0-7 *	9.2	15	42	59	37	4
**Eosino(#)**	0-480 *	797	1704	8442	13870	3480	230
**Baso(%)**	0-3	0.7	2	4	0	0	0
**PLT(/μl)**	155-347	677	679	528	467	370	160
**IgA(mg/dl)**	110-410	625	ND	511	ND	ND	ND
**IgG(mg/dl)**	870-1700	1301	ND	1323	ND	ND	ND
**IgM(mg/dl)**	33-190	60	ND	63	ND	ND	ND
**IgE(U/ml)**	10-350	ND	ND	428	501	ND	ND
**CRP(mg/dl)**	0-0.3	22.25	12.92	6.2	4.6	1.9	0.1

Abbreviations: Ref., reference; OPD, out-patient department; ND, not done.

#, Eosinophil counts.

* The normal range of eosino % and # in our hospital. The range of former's was 0-10% (0-960/μl).

## Method

Plasma was obtained from the patient three times during the course of observation and was stored at -80 degree. OPN & GAL-9 were measured by enzyme-linked immunosorbent assays (ELISA) as described previously [4]. The levels of sIL-2R, histamine and eotaxin-3 were measured also by ELISA (Cell free N IL-2R, Kyowa Medex, Japan; EIA histamine, Immunotech, France; Human CCL26/Eotaxin-3, R&D, Minneapolis). sIL-2R and eosinophils were measured 14 times during the hospital observations.

The cut-off points of the OPN, GAL-9, sIL-2R, histamine, eotaxin-3 and eosinophil counts were 820 ng/ml, 46 pg/ml, 519 U/ml, 0.18 ng/ml, 86 pg/ml and 480/μl respectively. The fold change values were calculated as observed value/cut-off point. The relative ratios to the eosinophil count were calculated as fold change values of each inflammatory marker/fold change of the eosinophil count.

## Results

**Figure 1:**
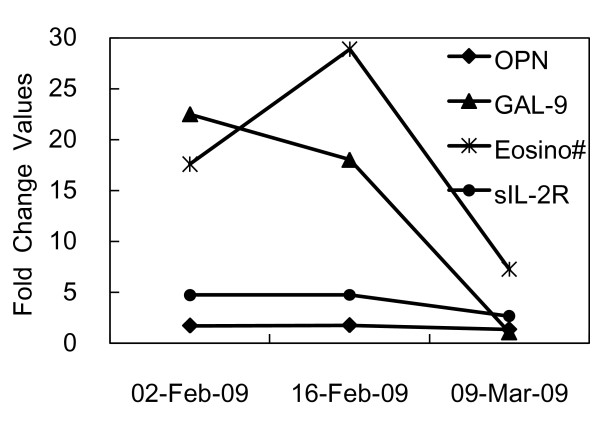
**The fold change values of plasma inflammatory molecules and eosinophil counts**. On Feb. 2, the eosinophil count (Eosino #) was already high (17.6 fold) and became the highest (35.5 fold) on Feb. 13 followed by a decrease after the cessation of insulin aspart & insulin aspart 30 mix on Feb. 13. OPN and sIL-2R were already elevated 1.7 fold and 4.7 fold, respectively on Feb. 2, but GAL-9 was elevated 22.5 fold more than the cut-off point. The elevations of OPN and sIL-2R were not changed on Feb. 16 but that of GAL-9 started to decrease. OPN and sIL-2R titers were still 1.4 and 2.7 fold higher on Mar. 9, but the GAL-9 level became normal.

On Feb. 2, the eosinophil count was already high (8,442/μl, 17.6 fold) and became higher (17,030/μl, 35.5 fold) on Feb. 13 followed by a decrease (13,870/μl, 28.9 fold) on Feb. 16 after the cessation of insulin aspart & insulin aspart 30 mix therapy on Feb. 13 (Figure 1). OPN and sIL-2R were already elevated 1.7 fold and 4.7 fold, respectively on Feb. 2, but the GAL-9 elevation was marked (22.5 fold). The elevations of OPN and sIL-2R were not changed on Feb. 16 but that of GAL-9 started to decrease. The levels of the eosinophil count, OPN and sIL-2R titers were still 7.3, 1.4 and 2.7 fold higher on Mar. 9, but the GAL-9 level became normal (Figure 1).

Furthermore, the presence of allergy was further supported by elevations of the eotaxin-3 and histamine levels. The levels of the former were 156 pg/ml (1.8 fold), 168 pg/ml (2.0 fold) and 120 pg/ml (1.4 fold) on Feb. 2, Feb. 16 and Mar. 9, respectively. The levels of the latter were 0.53 ng/ml (2.9 fold), 0.95 ng/ml (5.3 fold) and 0.65 ng/ml (3.6 fold) on the same days as above. The profiles of these two markers were similar to that of OPN (Figure 1).

**Figure 2:**
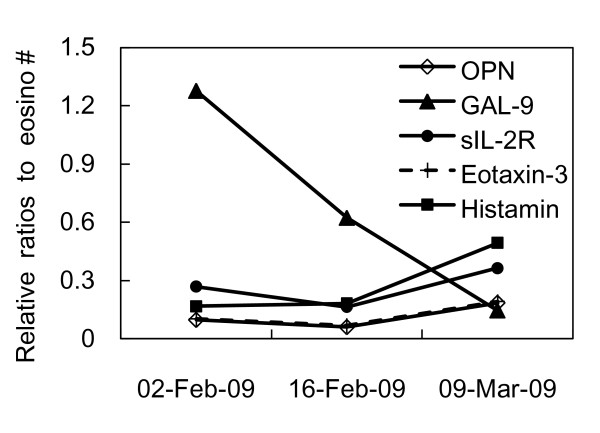
**The relative ratios of inflammatory markers to the eosinophil counts**. The profile of the relative ratios to the eosinophil counts for GAL-9 was very different from those of other markers. The relative ratios were calculated as the fold change value of each inflammatory marker/fold change of the eosinophil count (Eosino#).

The levels of inflammatory markers against the eosinphil counts are shown using the fold change in Figure 2. The profile of the relative ratios against the eosinophil counts in GAL-9 was very different from those of the other markers (Figure 2).

**Figure 3:**
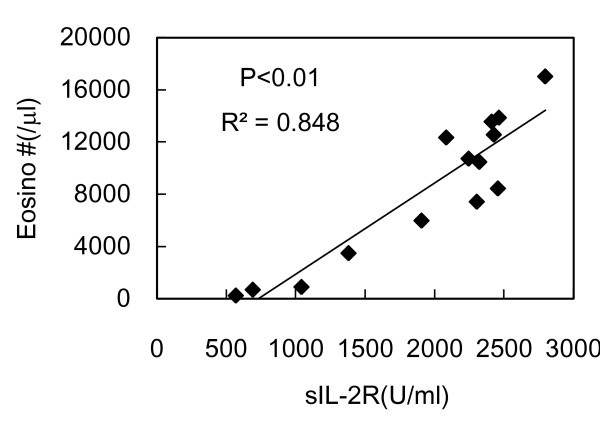
**The levels of sIL-2R showed a strong correlation with the eosinophil counts**. The closest association was found between sIL-2R and the eosinophil counts (Eosino#) (p < 0.01, Spearman rank correlation test).

In addition, the closest association was found between sIL-2R and the eosinophil count (p < 0.01, Spearman rank correlation test) (Figure 3).

## Discussion

The cause of the preceding fever in this patient was not known, but this event was not the cause of the eosinophilia because the count was normal before insulin was given. The decrease of the eosinphil count and total IgE after the cessation insulin led us to diagnose him as insulin allergy. Five inflammatory molecules which could be associated with eosinophilia were studied in this patient and we found elevations of the plasma levels of OPN and GAL-9 for the first time. The elevation of GAL-9 was marked (22.5 fold) and became normal within one month, although moderately high levels of eosinophil count (7.3 fold), OPN (1.4 fold), sIL-2R (2.7 fold), eotaxin-3 (1.4 fold) and histamine (3.6 fold) continued to be observed. Both eosinophils and mast cells are major effecter cells in acute allergic responses. And mast cells have been reported to synthesize OPN, which augments IgE-mediated degranulation and the migration of mast cells [9]. The increase of sIL-2R indicates T cell activation as well [10], and both the levels of OPN and sIL-2R did not become normal after the cessation of insulin. GAL-9 is also known to be expressed by human mast cells [11]. BAL fluid of patients with eosinophilic pneumonia contained high levels of GAL-9 and the levels were correlated with both the eosinophil count and eotaxin [12]. The anti-inflammatory activity of GAL-9 was implicated because it suppresses the release of mediators including histamine from mast cells by its binding to IgE [13]. In addition, GAL-3 has also been studied in eosinophilia, and the GAL-3 expression by eosinophil cells supports the cell adhesion to VCAM-1 and integrin and rolling to the site of inflammation [14]. However, another study showed that GAL-3 decreases the gene expression of IL-5 in an eosinophil cell line in vitro [15]. More detailed analyses of the galectin family in allergic conditions will be necessary.

In this study, the marked increase and swift decline of GAL-9 may suggest that it could reflect the activation of mast cells more accurately than sIL-2R and OPN, both of which could also reflect T cell and eosinophil cell activation. However, due to the limited patient samples, we could not show statistical correlations between each inflammatory marker in this study. Therefore, we propose that GAL-9 and OPN play roles in eosinophilia and the GAL-9 level could reflect the allergic conditions more accurately.

## Competing interests

The authors declare that they have no competing interest.

## Authors' contributions

HC-Y measured the levels of plasma osteopontin and wrote the article, and SB and URS analyzed the data of OPN and Gal-9. Drs. YA and HS analyzed clinical data. TA and MH measured the levels of plasma galectin-9. TH is responsible for all the work. All authors agreed to the final version of the manuscript.

## Consent statement

Written informed consent was obtained from the patient for publication of this case report and accompanying images. A copy of the written consent is available for review by the Editor-in-chief of this journal.
